# Seroprevalence and Associated Risk Factors of *Leptospira interrogans* Serogroup *Sejroe* Serovar *Hardjo* in Dairy Farms in and around Jimma Town, Southwestern Ethiopia

**DOI:** 10.1155/2021/6061685

**Published:** 2021-09-17

**Authors:** Garoma Desa, Yosef Deneke, Feyissa Begna, Tadele Tolosa

**Affiliations:** ^1^National Institute for the Control and Eradication of Tsetse Fly and Trypanosomosis, AkakiKaliti Sub-City P.O. Box 19917, Addis Ababa, Ethiopia; ^2^Jimma University, School of Veterinary Medicine, Jimma, Ethiopia

## Abstract

A cross-sectional study was conducted on selected dairy farms in and around Jimma town, Oromia, southwestern Ethiopia from November 2019 to May 2020 to determine the seroprevalence of *Leptospira interrogans serogroup Sejroe serovar Hardjo* (*L. hardjo*). Furthermore, information was gathered on individual animal and herd level by using pretested semistructured questionnaire to assess associated risk factors. A stratified and simple random sampling procedure was used for the selection of dairy farms and individual animal's, respectively. Indirect enzyme-linked immunosorbent assay (I-ELISA) was used in this study to detect antibody against *L. hardjo*. Out of 384 animal's sera, 94 animals were seropositive against *L. hardjo* antibodies. From 77 dairy farms selected for the study, 57 of them were distinguished as positive for *L. hardjo*. The overall seroprevalence of leptospirosis caused by *L. hardjo* was 24.48% (95% CI: 20.18%–28.78%) and 74.03% (95% CI: 64.23%–83.82%) at individual animal and farm level, respectively. The result of multilogistic regression analysis revealed that management system (*p* < 0.05; OR = 4.25 (95% CI: 2.31–7.82)), hygienic status of the farm (*p* < 0.05; OR = 0.35 (95% CI: 0.20–0.61)), age of animals (*p* < 0.05; OR = 8.30 (95% CI: 1.87–36.89)), history of abortion (*p* < 0.05; OR = 8.37 (95% CI: 1.73–40.42)), herd size (*p* < 0.05; OR = 2.32 (95% CI: 1.17–4.61)), and access of rodents to the farm (*p* < 0.05; OR = 0.17 (95% CI: 0.03–0.86)) were significantly associated with the occurrence of *L. hardjo* infection. However, breed, parity, and introduction of new animals to the farm were insignificantly associated (*p* > 0.05). Management system of the animal, hygienic status of the farm, herd size, age of animals, previous history of abortion, and access of rodents to the farm were identified as potential risk factors of *L. hardjo* disease occurrence. Thus, limiting rodents contact with cattle and their feed and water as well as good sanitary practices and husbandry management should be undertaken.

## 1. Introduction

Leptospirosis is a widespread disease of animals and also a zoonosis of worldwide distribution [1]. The disease has worldwide distribution due to the large spectrum of mammalian hosts that harbor and excrete the agent from their renal tubules [2]. The central point on the epidemiology of leptospirosis is the state of the renal carrier, the animal that has its renal tubules colonized by leptospirae, which in turn are excreted in the urine contaminating the environment [3]. Leptospirosis in cattle has important economic effects on the infected farms, resulting in reproductive losses due to infertility, abortions, stillbirths, weak offspring, and decreased milk production and growth rates [4].

Risk factors for cattle leptospirosis may include herd size, stocking density and herd management, grazing in areas shared with other infected cattle, pig, or sheep, presence of contaminated water sources, use of an infected bull, and age of the animals [5]. The core determinants of transmission of leptospiral infection are the presence of carrier animals, suitability of the environment for the survival of leptospires, and interaction between man, animals, and environment [6].

Diagnosis of leptospirosis depends on the samples available and temporal stage of the illness [7]. Serology is the most frequently used diagnostic approach for leptospirosis [8]. ELISA is one of the most widely used bioanalytical methods, where an antigen-antibody reaction occurs and the analyte of interest is detected by an enzyme reporter system [9]. It is characterized by high sensitivity and specificity compared to the microscopic agglutination test (MAT), the gold standard technique. Unlike MAT, ELISA can differentiate between individual immunoglobulin classes and therefore can be used to detect infections in early stages as well as older infections [7]. In indirect ELISA, samples to be analyzed for a specific antigen are adhered to the wells of a microtiter plate, followed by a solution of non-reacting protein such as bovine serum albumin (BSA) to block any areas of the wells not coated with the antigen [10].

Understanding the epidemiological features of leptospirosis is a critical step in designing interventions for reducing the risk of the disease transmission [11].

In Ethiopia, leptospirosis is a relatively unknown disease although already reported to occur in domestic animals.

Jimma zone has been known to be an area where forest coffee exists, and it has also humid environment with lots of cattle population. Even though previous study conducted by Yimer et al. [12] in Ethiopia confirmed the existence of *Leptospira* spp. in animals and humans, nothing has been known about the prevalence of the disease in the study settings. Hence, this study will be conducted to determine the serological prevalence of *L. hardjo* in selected dairy farms in and around Jimma town for the first time. Therefore, this study could complement the paucity of information about seroprevalence and risk factors associated with the occurrence of *L. hardjo* in dairy farm animals which are found in and around Jimma town in particular and also contribute to the government strategy to tackle the five zoonotic diseases including leptospirosis in the country at large.

## 2. Objectives

1. The objective is to determine the seroprevalence and associated risk factors of *Leptospira interrogans serogroup Sejroe serovar Hardjo* (*L. hardjo*) in dairy farms in and around Jimma town, southwestern Ethiopia.

## 3. Materials and Methods

### 3.1. Description of the Study Area

**Figure 1:**
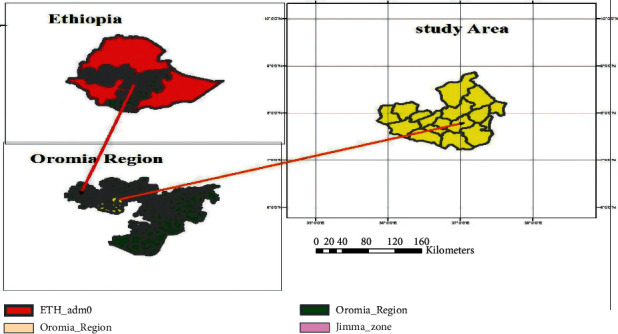
Map of the study area. Source: GIS 2019.

The study was conducted in and around Jimma town (Figure 1) of selected dairy farms from November 2019 to May 2020 in Jimma zone of Oromia regional state, at a distance of 355 km from Addis Ababa, the capital city of the country, southwestern Ethiopia. The area was located between 7° 41″ N latitude and 36° 50″ E longitudes and has an altitude of 1704 m. a. s. l. The climate of the area is a tropical humid climate characterized by heavy rainfall which ranges from 1200 to 2000 mm per annum. With the annual minimum and maximum temperature ranges from 6°C and 31°C, respectively, the overall average temperature is approximately 18.5°C. The agricultural production system in the area is mixed crop and livestock production system. Despite that the area is well known for its coffee production, still livestock production is one of the most important agricultural activities as well [13].

The zone is one of the largest owners of livestock populations in Ethiopia with an estimated population of 2,212,962 cattle, 866,561 sheep, 457,311 goats, 96,782 horses, 17,644 mules, 77,767 donkeys, 1,951,129 poultry, and 546,722 pieces of beehives [14].

### 3.2. Study Animals and Their Management

The target study population comprised apparently healthy animals of dairy farms that were managed under intensive and semi-intensive production systems. According to the criteria of Zuberbuhler et al. [15], management systems were classified as semi-intensive management system which includes all animals that are kept both indoor and outdoor while intensive management system covers all animals which were kept in closed housing system and feed concentrate as well as mixed feed. The cattle under study comprised exotic and local indigenous Zebu cattle of female animals with different age group greater than six months.

### 3.3. Study Design

A cross-sectional study was carried out, and a pretested semistructured questionnaire survey was conducted to collect data on associated risk factors in the study area.

### 3.4. Sample Size Determination and Sampling Technique

Sample size calculation was based on 50% prevalence assumption (since there was no study on *L. hardjo* in the area), 95% CI and *d* = 0.05 precision [16].

(1) $$\begin{array}{c} n=z^{2}\frac{\left(P\;\exp\right)\left(1-P\;\exp\;e\right)}{d^{2}}=\frac{{\left(1.96\right)}^{2}\left(0.5\right)\left(1-0.5\right)}{{\left(0.05\right)}^{2}}=384, \end{array}$$

where *n* = sample size, *z* = confidence statistic, *P*  exp = expected prevalence, and *d* = desired absolute precision.

Therefore, the sample size calculated was 384 cattle.

The sampling frame and sampling strategy were determined as follows.

A list of dairy cattle farms were obtained from official records maintained by Jimma Urban Agriculture and Natural Resource Office and list of animals from dairy farm owners. Based on the number of animals, farms were divided into three categories; small scale (≤10 heads of cattle), medium scale (>10–20 heads of cattle), and large scale (>20 heads of cattle) [17]. A stratified random sampling procedure was used for selection of dairy farms and study animals were selected by simple random sampling method (lottery method) depending on their ear tag. Based on its representativeness 11, 5, and 3 animals were sampled from large-, medium-, and small-scale farms, respectively.

### 3.5. Data and Sample Collection

#### 3.5.1. Questionnaire Survey

A pretested semistructured questionnaire was designed to collect information on factors that are believed to influence the spread and prevalence of *L. hardjo* infection at individual animal and farm level. The questionnaire was presented to the farmers by considering the general signs of the disease. Open and closed ended questions were used among the farm owners whose animals were sampled. Accordingly, 77 farm owners were interviewed for associated risk factors. The following data was collected on individual animal attributes: breed, age, parity, herd size, hygienic status of the farm house and management system. Based on its biological relevance, age is grouped into three categories: 0.50–<3years, 3–6years, and >6years according to their birth date records and dental eruption [18].

Besides, information on farms such as herd size, access of rodents to the farm, management system, history of abortion, hygienic status of the farm house and introduction of new animals to the farm (replacement heifers) (yes/no) was collected. Hygienic status of the farm house was categorized as clean/not clean based on manure disposal, drainage and barn ventilation while access of rodents to the farm was categorized as present/absent based on the presence/absence of animal feed storage (magazine) near to the farm and response of the respondents.

#### 3.5.2. Blood Sample Collection Procedures

Animals were restrained by animal handlers, and approximately 10 ml of blood sample was collected from the jugular vein of each animal using vacutainer tubes with 18–20 gauge hypodermic needles after cleaning the area with alcohol. Each sample from each animal was labeled by using codes describing the specific animal and farm. Corresponding to each sample, age and breed of every animal and other risk factors contributing to the occurrence of *L. hardjo* were collected and registered on a separate case book. The samples were then transported to Jimma University Veterinary Microbiology laboratory, School of Veterinary Medicine by using cool box.

Then, blood sample was kept overnight at room temperature to allow clotting. At the next morning, clearly separated serum of approximately 2 ml was decanted to the cryovials to which identification was coincided. The obtained sera were stored at −20°C until transported to National Veterinary Institute, and indirect enzyme-linked immunosorbent assay (indirect ELISA) was performed.

### 3.6. Laboratory Testing Procedures (Serology)

#### 3.6.1. Indirect Enzyme-Linked Immuno Sorbent Assay (I-ELISA)

The PrioCHECK *Leptospira interrogans serogroup Sejroe serovar Hardjo* (*L.hardjo*) antibody (Ab) is an indirect ELISA and detects Ab against *L. hardjo* in cattle. The test was performed as described by Scolamacchia et al. [19] and according to the manufacturer's recommendations. First, 100 *µ*l of ELISA buffer was dispensed to all wells of the test plate by using multichannel pipette, and the test plate was sealed by plastic plate sealer and then incubated for 1hour at 37°C. After the recommended stay, the ELISA buffer was discarded, and the test plate was washed six times with washing solution and then dried. Then, the three reference serums (positive control, negative control, and weak positive control) were diluted at 1 : 20 dilution, and 10 *µ*l of test sera was diluted in 190 *µ*l of ELISA buffer. 100 *µ*l of ELISA buffer was dispensed to wells *A*1 and *B*1 of the test plate (blanks). 90 *µ*l of ELISA buffer was dispensed to wells *C*1 to *H*1.

Then 10 *µ*l of 1 : 20 diluted reference serum 1 (positive control) was dispensed to wells *C*1 and *D*1. 10 *µ*l of 1 : 20 diluted reference serum 2 (negative control) was dispensed to wells *E*1 and *F*1. 10 *µ*l of 1 : 20 diluted reference serum 3 (weak positive control) was dispensed to wells *G*1 and *H*1. 90 *µ*l of ELISA buffer was dispensed and then again 10 *µ*l of diluted test sera was dispensed on each wells of test plate except control wells. Then, the test plate was sealed, shaken gently, and incubated for 1hour at 37°C. After 60minutes stay, the content was discarded, and the test plate was washed six times with washing solution and dried again.

Thereafter, 100 *µ*l of diluted conjugate solution was dispensed on each well, and the test plate was sealed and incubated for 1hour at 37°C for the third round after which the content was discarded and the test plate was washed six times with washing solution. Next, 100 *µ*l of the chromagen (TMG) substrate was dispensed to all wells and incubated for 15 minutes at room temperature. After 15 minutes, 100 *µ*l of stop solution was added to the wells, and the test plate was agitated to mix the content of the wells. Then, color change was observed, and the optical density (OD) of the wells was measured by ELISA reader at 450 nm within 15 minutes of stopping color development. The mean OD_450_ value of the blank wells (*A*1 and *B*1) and corrected OD_450_ value of all samples were calculated. Then, percentage positivity (PP) was calculated by the following formula:

(2) $$\begin{array}{c} \text{PP}=\left[\frac{\text{corrected }{\text{OD}}_{450}\text{ test sample}}{\text{corrected }{\text{OD}}_{450}\text{ reference serum }1}\right]\times 100. \end{array}$$

Finally, serum samples with PP of <20%, 20–45%, and >45% were interpreted as negative, inconclusive (antibodies may be present), and positive for *L. hardjo*-specific antibodies, respectively.

### 3.7. Data Management and Analysis

Data obtained from questionnaire survey and laboratory results were recorded, stored in Microsoft Excel and transferred to Stata version 12 statistical software for analysis. Data were coded and analyzed using descriptive and analytical statistics as appropriate. All of 384 samples were tested for *L. hardjo* by using I-ELISA. Two epidemiological parameters were generated, namely, individual animal seroprevalence and farm level prevalence. Individual animal seroprevalence was calculated by the number of positive animals divided by the total number of animals tested. Similarly, herd level prevalence was calculated by the number of positive farms divided by the total number of farms screened. Associations between outcome (*L. hardjo* seropositivity) and explanatory variables (risk factors) for all units of analysis were investigated by using binary logistic regression model. The strength of the association between outcome (*L. hardjo* seropositivity) and explanatory variables was assessed using the adjusted odds ratios (OR). Univariate logistic regression analysis was used to select the individual explanatory variable that may predict the outcome variable in the model. All risk factors that had noncollinear effect and *p*value of ≤0.25 in the univariable logistic regression analysis were subjected to multivariable logistic regression analysis to control the effect of confounding in the model.

## 4. Results

### 4.1. Questionnaire Survey

There were 169 dairy farms (28 large, 68 medium, and 73 small scale) in and around Jimma town with a total cattle population of 2,261. Accordingly, 77 farm owners were interviewed for associated risk factors. Out of the total, 51 (66.23%) and 26 (33.77%) respondents practice intensive and semi-intensive management system, respectively. Among the interviewed owners 12 (15.58%), 30 (38.96%), and 35 (45.46%) of them manage large, medium and small scale farm, respectively. Generally, the frequency distribution of management system, farm scale (size), access of rodents to the farm, history of abortion, hygienic practice of farm house and introduction of new animal to the farm is summarized in Table 1.

### 4.2. Overall Seroprevalence

#### 4.2.1. Individual Animal Level Seroprevalence of *L. hardjo*

Out of 384 sera samples, 94 (24.48%; 95% CI: 20.18–28.78%) were seropositive against *L. hardjo*-specific antibodies. According to univariable logistic regression analysis of risk factors associated with *L. hardjo* seropositivity at individual animal level (Table 2) age, hygienic status of the farm, management system, and herd size were significantly associated (*p* < 0.05) with seropositivity in the study area. But breed and parity were not significantly associated (*p* > 0.05).

#### 4.2.2. Farm Level Prevalence of *L. hardjo*

Out of 77 farms included in the study, 57 (74.03%; 95% CI: 64.23%–83.82%) of them were positive for *L. hardjo*-specific antibodies. In this study, farms with semi-intensive management system have significantly (*p* = 0.004) higher prevalence (96.15%; 95% CI: 88.76–103.55%) than intensively managed farms (62.75%; 95% CI: 49.48–76.01%). Similarly, the farm level univariable logistic regression analysis revealed that history of abortion, hygienic status of the farm house and access of rodents to the farm were found to be strongly associated with the farm positivity to *L. hardjo* (*p* < 0.05) while herd size and introduction of new animals to the farm showed insignificant association (*p* > 0.05) with *L. hardjo* disease occurrence (Table 3).

#### 4.2.3. Potential Risk Factors

Variables with a *p* ≤ 0.25 in the univariable analysis were included in the final multivariable logistic regression model. Accordingly, age, breed, parity, management system, hygienic status of the farm house, and herd size from individual animal level risk factors were included in the final logistic regression model. Concerning farm level risk factors management system, herd size, access of rodents to the farm, history of abortion, and hygienic status of the farm house were selected for final model. In the final analysis, animals seropositivity was influenced more by management system, hygienic status of the farm house, herd size, age and previous history of abortion (Table 4). Access of rodents to the farm was also significantly associated with *L. hardjo* seropositivity. Thus, multivariable logistic regression analysis depicts that *L. hardjo* seropositivity was found to be 8.30 (95% CI 1.87–36.89) times higher among the animals of age group >6years than age groups of 0.5–<3 years. Seroprevalence, recorded for cattle, in large (35.11%) and medium (21.58%) herd size revealed a statistically significant variation (*p*< 0.05) with the odds ratio of seropositivity of 2.32 and 2.18 times more likely to be infected with *L. hardjo*, respectively, than animals of small herd size. Seropositivity of *L. hardjo* was significantly associated (*p*= 0.008) with farms having previous history of abortion than those did not have. Similarly, seropositivity of the organism was significantly associated (*p*= 0.001) with animals managed semi-intensively than intensively.

## 5. Discussion

In the present study, a total of 384 serum samples were collected from selected dairy farms in and around Jimma town for the detection of anti-*Leptospira interrogans serogroup Sejroe serovar Hardjo* (*L. hardjo*) antibody by indirect ELISA. As a limitation of the study, indirect ELISA needs an extra incubation step in the procedure and cross-reactivity might also occur with the secondary antibody, resulting in nonspecific signal.

The results revealed that a total of 94 sera were positive (animal level seroprevalence 24.48% (95% CI: 20.18%–28.78%)). This result was in agreement with the findings of Odontsetseg et al. [20] (23.50%) in Mongolia, Schoonman and Swai [21] (30.30%) in Tanzania, Gamage et al. [22] (20.30%) in Sri Lanka and Subharat et al. [23] (27.4%) in Australia. Similarly, the present finding was in congruence with results of previous studies by Taddei et al. [24] (19.30%) in unvaccinated animals of Colombian dairy farm, Ismail et al. [25] (26.25%) in Jordan, Tabatabaeizadeh et al. [26] (19.10%) in Iran, Ngbede et al. [27] (25% and 23.90%) in different dairy farms of Zaria (Nigeria), Balamurugan et al. [28] (23.68%) in Chhattisgarh of India, Ismail et al. [25] (28.75%) in Jordan and Shilpa *et al.* [29] (19.92%) in Nagpur of Indian dairy farms.

In contrast, by far higher seroprevalence of *L. hardjo* has been reported in some countries like 88.20% in Mexico [30], 87% in India [31], 45.60% in New Zealand [32], and 42.27% in Pakistan [33]. However, lower results were recorded in United States (15%) [34], Urmia of Iran (8.38%) [35], various Indian states such as Punjab (3.70%), Gujarat (13.50%), Haryana (4.46%), Telangana (4%), Jharkhand (10%) [28], Lalitpur, Nepal (3.75%) [36], and in Central and Northern Madagascar (13.90%) [37]. There was also another study by Ramyasree et al. [38] who reported lower findings (12.98%) from dairy farms of Andhra Pradesh, India.

This great variation in the seroprevalence rates of *L. hardjo* over the world is most likely due to variation in geographical location, management systems, husbandry practices, different breeds of animals, levels of natural immunity and disease resistance among studied populations [21, 33].

In this study, prevalence of 74.03% (95% CI: 64.23%–83.82%) *L. hardjo* infection was found at farm level which coincides with the finding of Webster and Macdonald [39] and Schafbauer et al. [37] in England and Central and Northern Madagascar, where farm level prevalence rate of *L. hardjo* figures 72% and 74% respectively. In contrast, it was higher than the farm level prevalence recorded in USA (42%) [40], Algeria (31.25%) [41], Spain (11%) [42], and Thailand (28.60%) [43] and lower than that of Ryan et al. [44] (82.29) in Irish, Campos et al. [45] (100%) in Brazilian, and Ismail et al. [25] (92.30%) in Jordanian dairy farms.

There was statistically significant association (*p* = 0.001; OR = 4.25; Chi^2^ = 31.94) between management system and seropositivity of *L. hardjo* in the present study. The analysis revealed that female animals raised in semi-intensive management system were significantly at higher risk of becoming seropositive to the infection. This result was in line with previous finding of Yatbantoong and Chaiyarat [43] in Thailand dairy farms where semi-intensively handled animals were significantly associated with *the L. hardjo* infection. This could be attributed to poor husbandry practices and to the fact that infected animals increase the risk of contaminating the environment during cograzing [46]. It is also acknowledged that sharing pasture increases the risk of *Leptospira* transmission as has been observed previously [45].

In this study, the seropositivity of *L. hardjo* is significantly associated (*p* = 0.001; OR = 0.35; Chi^2^ = 13.05) with unclean farm houses and their animals than those categorized as clean both at farm and individual animal levels. This finding was in line with the previously reported work of Ismail et al. [25] who reported significant difference in disease seropositivity between clean and unclean farms. This goes along with poor hygiene and sanitation practices in some dairy farms with overcrowded populations [33]. It is also known that poor sanitation is among the core determinants that favor transmission of leptospirosis [47, 48] and sanitation of animal habitat governs source of the disease [4].

The multivariable logistic regression analyses showed that older cows were 8 times more likely to be seropositive compared to younger animals (*p* = 0.005; OR = 8.30; Chi^2^ = 8.35). The present finding with respect to age wise prevalence is in accordance with the earlier study of Salas [49], Leahy et al. [50] and Prescott et al. [51] who observed more seropositivity in older cattle. It also agrees with the work of Behera et al. [52] who reported increased detection of anti *L. hardjo* antibodies in age groups o >5 years than in those <6 months old age group in Odisha and West Bengal, India. According to Black et al. [53], age of cattle was statistically significantly associated with infection by *Leptospira* spp. This result is also in accordance with previous findings in Iran and other countries where seropositivity to leptospirosis increases as the animals age increases [54, 55].

This could be attributed to the duration of exposure and persistence of the antibodies in the aged animals to the pathogen [56]. Actually, seroprevalence in young domestic cattle has been reported to be lower than that in older domestic cattle [57]. However, it differs from previous study reported in Turkey where age was not a significant factor [58, 59] in Trinidad.

In the current study, Previous history of abortion at farm level was also associated with high risk of disease occurrence with *L. hardjo* (*p* = 0.008; OR = 8.37; Chi^2^ = 12.75). This result is in line with the previous work conducted by Balamurugan et al. [28] who reported significant association between previous history of abortion and occurrence of the disease. It is also in agreement with the finding of Ismail et al. [25] who indicated significant number of farms with previous history of abortion found infected with *L. hardjo*. This can be viewed as an evidence of the widespread of *L. hardjo* as a cause of bovine abortion in the studied population. Additionally, in some circumstances, abortion is the principal manifestation of leptospirosis due to *L. hardjo* which is the major cause of abortion in cattle [60]. In contrast, Yatbantoong and Chaiyarat [43] reported insignificant association between previous history of abortion and occurrence of the disease.

Animals from large herd size were significantly at higher risk of becoming seropositive to *L. hardjo* infection (OR = 2.32; *p* = 0.02; chi^2^ = 13.30). This is in congruence with the previous results of Bahaman et al. [61] in west Malaysia, Tabatabaeizadeh et al. [26] in Iran, Benseghir et al. [41] in Algeria, and Yatbantoong and Chaiyarat [43] in Thailand. The reason for this association most likely relates to the increased risk of exposure, transmission and persistence of infection in larger herds [62, 63]. A positive association between herd size and the presence of positive animals has been reported previously for *L. hardjo* infection in cattle [64, 65]. Herd size of animals has also been shown to be risk factors for *Leptospira* infection [44, 66].

According to Mathiase and Levett [67], population size of the farm is among the main factors that determine the source of leptospirosis. Majority of the large dairy farms demonstrated a high prevalence of *Leptospira* infection reported by Bahaman et al. [61]. The hygienic measurement and sanitation facilities in large scale dairy farm are poor as compared to small scale dairy farm and overcrowded population helps in spreading the infection rapidly and these might be potential risk factors for higher prevalence of leptospirosis.

In this study, there is statistically significant difference (*p* = 0.03; OR = 0.17; Chi^2^ = 10.94) in *L. hardjo* infection between farms having access of rodents and those did not have. This finding is in accordance with the previous work conducted in Puente Piedra, Mexico, by Platts-Mills et al. [68] who concluded that availability of rodents around the dairy farm might be one of the reasons for high prevalence of the disease. This might be due to the fact that rodents are considered as the major reservoir of leptospires [4].

Athanazio et al. [69] also indicated urine of animals, mainly rodents, which may become asymptomatic carriers, constitute the reservoirs of *Leptospira* in nature.

In contrast to the findings of Yatbantoong and Chaiyarat [43] who reported significant association between introduction of new animals to the farm and disease occurrence, in this study introduction of new animals to the farm was insignificantly associated (*p* = 0.68; OR = 0.77; Chi^2^ = 0.17) with *L. hardjo* disease occurrence. This is due to the fact that the newly introduced animals to the farm might be free of *L. hardjo* in the current study.

Statistically, there is no significant difference (*p* = 0.19; OR = 1.45; Chi^2^ = 1.71) between different breeds against *L. hardjo* during the present study. This is in accordance with the work of Parvez et al. [33] conducted on *L. hardjo* in dairy cattle of Chittagong, Bangladesh, who reported insignificant association. Similarly, it is also in agreement with previous findings of Rajala et al. [70] in Tajikistan, Benseghir, et al. [41] in Algeria, and Ngwa et al. [71] in Cameroon who obtained insignificant difference between different breeds against *L. hardjo* infection. Contrastingly, Bahaman et al. [61] reported a significant difference between different breeds against seropositivity of the infection that showed the drought masters had the highest prevalence whilst the Kedah-Kelantan (an indigenous breed) had the lowest prevalence of leptospiral infection.

On attempt to know the influence of parity, statistically there was no significant difference (*p* = 0.17; OR = 1.65; Chi^2^ = 3.29) between various parity of the cows and seropositivity of *L. hardjo*. This result is in accordance with the previous study conducted on seroprevalence and risk factors of *L. hardjo* infection in dairy cows in Jordan by Ismail et al. [25] who reported insignificant association between parity and seropositivity of the infection. In this study, farm size was also insignificantly associated (*p* = 0.20; OR = 2.95; Chi^2^ = 4.20) with *L. hardjo* infection at farm level which is in agreement with previous finding of Parvez et al. [33] in dairy cattle of Chittagong, Bangladesh.

## 6. Conclusion and Recommendations

An overall seroprevalence of 24.48% and 74.03% *Leptospira interrogans serogroup Sejroe serovar Hardjo* was observed at individual animal and herd level respectively in present study area. Management system of the animal, hygienic status of the farm, herd size, age of animals, previous history of abortion and access of rodents to the farm were identified as potential risk factors of *Leptospira interrogans serogroup Sejroe serovar Hardjo* disease occurrence. On the other hand, breed, parity and introduction of new animals to the farm were insignificantly associated with seropositivity against *L. hardjo* in this study. The current finding indicates that leptospirosis caused by *L. hardjo* was highly prevalent in selected dairy farms in and around Jimma town, southwestern Ethiopia.

Therefore, we recommend the implementation of hygienic practices in farms to reduce the spread of infection, as well as the use of vaccination in animals at risk. We also recommend wide surveys in animals all over the country to assess the real prevalence of leptospirosis in Ethiopia.

## Figures and Tables

**Table 1:** Owners response on dairy farm information with their frequency and percentage.

Parameters	Categories	Frequency	Percentage (%)
Herd size	Large	12	15.58
	Medium	30	38.96
	Small	35	45.46

Access of rodents to the farm	Present	34	44.16
	Absent	43	55.84

History of abortion	Present	41	53.25
	Absent	36	46.75

Hygienic status of farm house	Clean	42	54.55
	Not clean	35	45.45

Introduction of new animal to farm	Yes	18	23.38
	No	59	76.62

Management system	Intensive	51	66.23
	Semi-intensive	26	33.77

**Total**		**77**	**100.00**

**Table 2:** Univariable logistic regression analysis of risk factors associated with *L. hardjo* seropositivity at individual animal level.

Risk factors	Categories	No tested	No. of positives	Preval. (%)	OR (95% CI)	*p* value
Age	0.5–<3years	162	28	17.28	Ref	
	3–6years	122	34	27.87	1.85 (1.05–3.26)	0.034
	>6years	100	32	32.00	2.25 (1.25–4.04)	0.007

Breed	Local	76	23	30.26	1.45 (0.83–2.53)	0.19
	Exotic	308	71	23.05	Ref	

Parity	0 parity	157	31	19.75	Ref	
	1–2	126	35	27.78	1.56 (0.90–2.72)	0.11
	3	49	13	26.53	1.47 (0.70–3.09)	0.31
	>3	52	15	28.85	1.65 (0.80–3.38)	0.17

Mgt. system	Intensive	282	48	17.02	Ref	
	S/intensive	102	46	45.09	4.00 (2.43–6.60)	0.001

Hyg. status	Clean	201	34	16.92	Ref	
	Not clean	183	60	32.79	0.42 (0.26–0.68)	0.001

Herd size	Small	114	18	15.79	Ref	
	Medium	139	30	21.58	1.47 (0.77–2.80)	0.244
	Large	131	46	35.11	2.89 (1.56–5.36)	0.001

**Total**		**384**	**94**	**24.48**		

OR = odds ratio, CI = confidence interval, Ref = reference.

**Table 3:** Univariable logistic regression analysis of risk factors associated with *L. hardjo* disease occurrence at farm level.

Risk factors	Categories	No. tested	No. of positives	Preval. (%)	OR (95% CI)	*p* value
Mgt. system	Intensive	51	32	62.75	Ref	
	S/intensive	26	25	96.15	7.25 (1.91–27.54)	0.004

Herd size	Small	35	22	62.86	Ref	
	Medium	30	25	83.33	2.95 (0.91–9.61)	0.72
	Large	12	10	83.33	2.95 (0.56–15.63)	0.20

Access of rodents	Present	36	33	91.67	0.13 (0.03–0.49)	0.003
	Absent	41	24	58.54	Ref	

History of abortion	Present	38	35	92.11	0.11 (0.03–0.42)	0.001
	Absent	39	22	56.41	Ref	

Hygienic status	Clean	42	25	59.52	Ref	
	Not clean	35	32	91.43	0.14 (0.04–0.52)	0.004

Introduction of new animals	Yes	18	14	77.78	0.77 (0.22–2.68)	0.68
	No	59	43	72.88	Ref	

**Total**		**77**	**57**	**74.03**		

OR = odds ratio, CI = confidence interval, Ref = reference.

**Table 4:** Multivariable logistic regression analysis of potential risk factors with *L. hardjo* seropositivity.

Risk factors	Categories	OR (95% CI)	*p* value (Chi^2^)
Age	0.5–<3years	Ref	
	3–6years	1.97 (0.69–5.60)	0.21
	>6years	8.30 (1.87–36.89)	0.005 (8.35)

Management system	Intensive	Ref	
	S/intensive	4.25 (2.31–7.82)	0.001 (31.94)

Hygienic status	Clean	Ref	
	Not clean	0.35 (0.20–0.61)	0.001 (13.05)

History of abortion	Present	8.37 (1.73–40.42)	0.008 (12.75)
	Absent	Ref	

Herd size	Small	Ref	
	Medium	2.18 (1.06–4.48)	0.03
	Large	2.32 (1.17–4.61)	0.02 (13.30)

Access of rodents to the farm	Absent	Ref	
	Present	0.17 (0.03–0.86)	0.03 (10.94)

OR = odds ratio, CI = confidence interval, Ref = reference.

## Data Availability

The data used to support the findings of this study can be received from the author on request.
